# Molecular Characterization of Non-H5 and Non-H7 Avian Influenza Viruses from Non-Mallard Migratory Waterbirds of the North American Flyways, 2006–2011

**DOI:** 10.3390/pathogens13040333

**Published:** 2024-04-17

**Authors:** Shahan Azeem, John Baroch, Deepanker Tewari, Kristy L. Pabilonia, Mary Killian, Birgit Bradel-Tretheway, Dong Sun, Sara Ghorbani-Nezami, Kyoung-Jin Yoon

**Affiliations:** 1 Department of Veterinary Microbiology and Preventive Medicine, Iowa State University, Ames, IA 50011, USA; sazeem@uvas.edu.pk (S.A.); dong.sun@zoetis.com (D.S.); 2 Institute of Microbiology, Faculty of Veterinary Science, University of Veterinary and Animal Sciences, Lahore 54000, Pakistan; 3 Wildlife Services, Animal & Plant Health Inspection Service (APHIS), United States Department of Agriculture (USDA), Fort Collins, CO 80526, USA; 4 Pennsylvania Veterinary Laboratory, Pennsylvania Department of Agriculture, Harrisburg, PA 17110, USA; dtewari@pa.gov; 5 Department of Microbiology, Immunology and Pathology, Colorado State University, Fort Collins, CO 80523, USA; kristy.pabilonia@colostate.edu; 6 National Veterinary Services Laboratories, Animal & Plant Health Inspection Service (APHIS), United States Department of Agriculture (USDA), Ames, IA 50010, USA; mary.l.killian@aphis.usda.gov; 7 Paul G. Allen School for Global Animal Health, Washington State University, Pullman, WA 99164, USA; bgbt107@yahoo.com; 8 Department of Veterinary Diagnostic and Production Animal Medicine, Iowa State University, Ames, IA 50011, USA

**Keywords:** ducks, phylogenetic analysis, North American flyways, surveillance

## Abstract

The surveillance of migratory waterbirds (MWs) for avian influenza virus (AIV) is indispensable for the early detection of a potential AIV incursion into poultry. Surveying AIV infections and virus subtypes in understudied MW species could elucidate their role in AIV ecology. Oropharyngeal–cloacal (OPC) swabs were collected from non-mallard MWs between 2006 and 2011. OPC swabs (*n* = 1158) that molecularly tested positive for AIV (Cts ≤ 32) but tested negative for H5 and H7 subtypes were selected for virus isolation (VI). The selected samples evenly represented birds from all four North American flyways (Pacific, Central, Mississippi, and Atlantic). Eighty-seven low pathogenic AIV isolates, representing 31 sites in 17 states, were recovered from the samples. All isolates belonged to the North American lineage. The samples representing birds from the Central Flyway had the highest VI positive rate (57.5%) compared to those from the other flyways (10.3–17.2%), suggesting that future surveillance can focus on the Central Flyway. Of the isolates, 43.7%, 12.6%, and 10.3% were obtained from blue-winged teal, American wigeon, and American black duck species, respectively. Hatch-year MWs represented the majority of the isolates (70.1%). The most common H and N combinations were H3N8 (23.0%), H4N6 (18.4%), and H4N8 (18.4%). The HA gene between non-mallard and mallard MW isolates during the same time period shared 85.5–99.5% H3 identity and 89.3–99.7% H4 identity. Comparisons between MW (mallard and non-mallard) and poultry H3 and H4 isolates also revealed high similarity (79.0–99.0% and 88.7–98.4%), emphasizing the need for continued AIV surveillance in MWs.

## 1. Introduction

The influenza virus, an enveloped RNA virus belonging to the *Orthomyxoviridae* family, is known to infect humans, mammals, and birds [1]. Viruses belonging to the genus *Alphainfluenzavirus* are of primary human and animal health significance. Avian influenza virus (AIV) or the influenza A virus (IAV) of birds is an Alphainfluenzavirus that can be further classified into subtypes based on the antigenic and genetic similarity of two surface glycoproteins: hemagglutinin (HA or H) and neuraminidase (NA or N). To date, 16 H and 9 N subtypes and their most combinations have been found in migratory waterbirds (MWs), making them the natural reservoir of AIVs [1,2]. The viruses can also be divided into two biotypes, low and highly pathogenic AIVs, based on their virulence in gallinaceous poultry [3].

Studying AIV infections and diversity in their natural host is paramount for understanding AIV ecology and epidemiology. Such information is crucial for geographic areas with a high concentration of poultry production facilities that overlap with the migratory routes or stopover points of wild birds due to the possibility of virus spillover from MW to domestic poultry [4,5]. Information on AIV subtypes circulating in MWs could serve as an early warning system for poultry producers, as some AIV subtypes are likely to mutate to highly pathogenic avian influenza viruses (HPAIVs) [1,6].

The emergence of HPAIV H5N1 in Asia in 1996 led to worldwide surveillance programs with the goal of early detection of HPAIV [7]. Migratory waterbirds, such as ducks, geese, gulls, and swans, serve as a natural reservoir for AIVs of all subtypes [1,6], which occasionally spill over to domestic poultry. Migratory waterbird’s unprecedented involvement in the global ecology of AIVs implies the possible co-circulation of both low pathogenic avian influenza viruses (LPAIVs) and HPAIVs, making them a serious threat to poultry [8]. Yet, recent HPAI outbreaks in Canada and the United States reaffirm that MWs can be symptomatically infected with and carry HPAIV [9]. These outbreaks have occurred with concomitant HPAIV outbreaks in commercial and backyard poultry flocks [10]. From 2006 to 2011, the United States developed and deployed a comprehensive surveillance program for AIVs in migratory wild bird populations [11,12]. This program, together with the Canadian and Mexican programs for AIV surveillance constituted the largest systematic wildlife disease surveillance program ever implemented [11]. Migratory waterbirds from all four administrative North American flyways (Pacific, Central, Mississippi, and Atlantic) were surveyed nationwide [12]. Despite the extensive design of this surveillance program, the overall program had two unresolved issues, which led to the focus of this research study. First, due to the significant economic impact of HPAIVs compared to LPAIVs, the primary focus of the program was the detection of HPAIVs, specifically H5N1 [13], leaving most LPAIVs poorly studied. Second, since dabbling ducks, in particular mallards (*Anas platyrhynchos*), are known to have the highest prevalence of AIVs, the surveillance focused on them [14,15], neglecting non-mallard MWs, which play an equally important role in AIV ecology.

The present study was conducted to assess the positivity rate, subtype diversity, and molecular characteristics of the LPAIV of non-H5/H7 subtypes in non-mallard MWs sampled during the 2006–2011 USDA’s AIV surveillance study [11,16]. The study also compared the positivity rate and subtypes of LPAIV in non-mallard MWs to those in mallard ducks and poultry during the study period. Furthermore, the most common subtypes of non-mallard LPAIV isolates were genetically compared with LPAIVs from mallard ducks and poultry during the same period. It was hypothesized that the study would provide a baseline for understanding species-wide virus positivity rates and AIV subtype diversity in various non-mallard MW species because of extensive and systemic sampling over five consecutive biological years, even though the sample set was not temporally contemporary.

## 2. Materials and Methods

### 2.1. Ethics Statement

Migratory waterbird surveillance activities were conducted in accordance with permitting agencies and/or with the consent of private landowners. Migratory waterbird capture and sampling were approved by the USA Fish and Wildlife Service (Permit Number MB124992) for high pathogenic avian influenza surveillance. Samples collected at hunter-check stations were collected through state and local officials and with the consent of participating hunters.

### 2.2. Sampling

Risk-based surveillance was used to stratify wild birds, first by flyway and then by species [11]. The samples were collected through the national migratory wild bird HPAIV surveillance program organized by the United States Department of Agriculture (USDA) and the Department of Interior in cooperation with numerous state agencies and collaborating laboratories [11]. The national surveillance program sampled 217,428 non-mallard migratory wild birds on a biological-year basis from 1 April 2006 to 31 March 2011. One or more of the following five strategies were used to collect samples: (1) investigating morbidity and mortality, (2) hunter–harvest sampling, (3) live-bird sampling, (4) sampling from sentinel species, and (5) environmental sampling comprising wild bird feces [17]. Both hatch-year (HY) and after-hatch-year (AHY) birds were included in the sampling. Of these samples, 2444 combined oropharyngeal–cloacal swabs were positive for the AIV matrix (M) gene by a real-time reverse transcription–polymerase chain reaction (rRT-PCR) but negative for H5 and H7 subtypes.

Of the AIV M gene-positive oropharyngeal–cloacal swabs, samples with cycle threshold (Ct) values equal to or lower than 32 were selected for this study. The final subset of M gene-positive samples meeting the selection criteria included 1158 oropharyngeal-cloacal swabs that represented 17 species of non-mallard MWs from 151 sites in 112 counties in 36 states. The majority of the present study’s samples (97.6%) were collected using hunter–harvest or live-bird sampling to maximize the randomness of samples. The remaining samples represented morbidity/mortality sampling. The sample selection was performed evenly from all four North American flyways: Atlantic, Pacific, Central, and Mississippi [11,18]. The contiguous 48 states span latitudes from roughly 25 degrees to 49 degrees north. The study included a limited number of samples from lower latitudes and wintering sites, including twenty-eight from New Mexico, three from North Carolina, two from California, two from Florida, one from Mississippi, and one from Utah. Samples were collected from the following species: American black duck, American green-winged teal, American wigeon, blue-winged teal, cinnamon teal, gadwall, greater snow goose, lesser scaup, lesser snow goose, mute swan, northern pintail, northern shoveler, ring-billed gull, ruddy turnstone, trumpeter swan, tundra swan, and wood duck.

The selected samples were tested by virus isolation (VI). All isolates were sequenced for M, HA, and NA and genes for subtyping and sequence comparison. Subtype diversity and molecular characteristics were compared among non-mallard, mallard, and poultry isolates during the same time period.

### 2.3. Virus Isolation

Virus isolation was conducted using specific-pathogen-free, 9- to 10-day-old embryonated chicken eggs using the allantoic route inoculation method [19]. Briefly, brain heart infusion (BHI) media containing each oropharyngeal–cloacal swab was vortexed and centrifuged at 1500× *g* for 15 min, and the resulting supernatant was collected for the isolated virus. An antibiotic–antimycotic solution was added to each supernatant at the following final concentration: penicillin G 2000 IU/mL, streptomycin sulfate 0.2 mg/mL, gentamicin sulfate 0.25 mg/mL, and amphotericin B 500 IU/mL [20]. The supernatants were incubated at ambient temperature for approximately 2 h prior to inoculation to inactivate possible contaminants [21]. Each supernatant was then inoculated to at least two eggs (0.1–0.2 mL/egg). The inoculated eggs were incubated at 37 °C in a humidified egg incubator for five days with daily monitoring for embryo death through candling. Eggs with dead embryos within 24 h post-inoculation were discarded. Allantoic fluid was harvested from all remaining eggs at the end of the 5-day incubation period, except ones with dead embryos from which allantoic fluids were harvested upon detecting embryonic death. The allantoic fluids were centrifuged at 1500× *g* for 15 min for clarification. Virus growth in embryonated eggs was confirmed by a hemagglutination assay, followed by rRT-PCR targeting the M gene of AIV [22,23]. One blind passage was made on all test-negative samples before being considered virus-negative.

AIV isolation data from mallard ducks were kindly provided by the National Wildlife Research Center, United States Department of Agriculture (USDA)/Animal and Plant Health Inspection Service (APHIS)/Wildlife Services (WS), in Fort Collins, CO, USA.

### 2.4. Data Analysis

The statistical comparisons of the VI success rates between the non-mallard and mallard MWs were performed using the proportion test (Z distribution) with a significance level set at 0.05. Minitab statistical package version 17 (Minitab Inc., State College, PA, USA) was used. Likewise, the positivity rate comparisons across flyways, sex, and age were also made using the same method. Descriptive statistics were used to compare the VI success rate among various non-mallard species.

### 2.5. Hemagglutination Assay

Fifty µL of 0.1 M phosphate-buffered saline (PBS) at pH 7.2 was added to each well of U-bottom 96-well plates, followed by the addition of 50 μL of allantoic fluid in the first well (i.e., 1:2 dilution) after thorough mixing, followed by serial two-fold dilutions (up to 1:1024). A negative control (PBS) and positive control (AIV) were included in the 11th and 12th wells, respectively. Fifty µL of 0.25% rooster red blood cells was added to all the wells. The plate was incubated for 30 min at ambient temperature. The titer of each isolate was read as the reciprocal of the highest dilution in which complete hemagglutination was observed and recorded as the hemagglutination unit (HAU) per 50 µL [22]. The endpoint was considered as 1 HAU.

### 2.6. Nucleotide Sequencing

AIV isolates were sequenced for M, HA, and NA genes to determine subtypes and conduct sequence comparisons [24]. The matrix gene was chosen because this gene is relatively conserved and is one of the most abundant genes of AIVs. Hemagglutinin and neuraminidase gene sequencing was performed for subtyping. The three genes were also used in phylogenetic analyses to trace the origin and relatedness of AIVs originating from different avian host species.

First, viral RNA was extracted using a spin column technique as per the manufacturer’s instructions (QIAamp Viral RNA Mini Kit, Qiagen, Valencia, CA, USA) [25]. Full-length M, HA, and NA genes were then amplified by RT-PCR using SuperScript™ III One-Step RT-PCR kit with Platinum^®^ Taq High Fidelity polymerase (Invitrogen, Carlsbad, CA, USA). The primer information was either already published [26,27,28,29,30,31] or provided in Table 1.

Whenever a less conspicuous DNA band was shown on agarose gel electrophoresis, a PCR reaction with Vent Polymerase (New England Biolabs Inc., Ipswich, MA, USA) was set up using the same primer pair that was used in the RT-PCR described above. The amplicon from the RT-PCR was used as a template [32].

All amplified PCR products were purified using either the QIAquick PCR Purification Kit (Qiagen, Valencia, CA, USA) or the QIAquick Gel Extraction Kit (Qiagen, Valencia, CA, USA). Then, resulting PCR amplicons along with sequencing primers were submitted to either the Iowa State University DNA Facility (Ames, IA, USA), Eurofins Lancaster Laboratories Environmental, LLC (Lancaster, PA, USA), or the University of California-Davis DNA Sequencing Facility (Davis, CA, USA) for sequencing. For M gene sequencing, two pairs of primers were used: one pair was the amplification primers used in the RT-PCR, and the other pair was internal sequencing primers [26]. The PCR amplicons of the HA gene were sequenced using the amplification primers and the internal subtype-specific sequencing primers already published [26,27,28,29,30,31] or those described in Table 1. If initial amplification attempts to obtain a full-length HA gene using Bm-HA1 and Bm-NS-890R were not successful, a 640 bp fragment from its 3′-end was amplified using HA-1134F and Bm-NS-890R [32]. This fragment’s sequence was then used in the BLAST^®^ tool available in GenBank^®^ (http://blast.ncbi.nlm.nih.gov/Blast.cgi) to identify the HA subtype. The remaining 1134 bp fragment at the 5′-end of the HA gene was then amplified using HA-1F and HA-1157R or subtype-specific amplification and sequencing primers (Table 1). To sequence the NA gene, the viral RNA was amplified using the Bm-NA-1 and Bm-NA-1413R primers to obtain the full-length NA fragment. The obtained PCR amplicons were sequenced using the amplification primers. Once the NA subtype was determined, subtype-specific internal primers were used to obtain the 5′ and 3′-ends. Since the NA gene of N3, N6, N7, and N9 subtypes could not be amplified using Bm-NA-1 and Bm-NA-1413R, subtype-specific primers were adopted and used for amplification and sequencing [26,33]. The sequences were submitted to GenBank and the following accession numbers were obtained: OQ985055, OQ985057, OQ985353, OQ985363, OQ985959, OQ986003, OQ987892—OQ987894, PP212034, PP212822—PP212832, PP258641—PP258662, PP264487—PP264521.

### 2.7. Phylogenetic Analyses

Sequence data were assembled and aligned using Lasergene (version 11, DNASTAR Inc., Madison, WI, USA) and BLAST^®^. Phylogenetic analyses were performed with the Clustal W method. The phylogenies were constructed using the maximum likelihood method and Tamura–Nei model using the Molecular Evolutionary Genetics Analysis software: MEGA X (version 10.2.2) [34]. The analyses focused on the most prevalent HA or NA subtypes identified during the study, while M gene sequences from all isolates were compared.

For sequence comparisons, the AIV sequences from mallards, which were obtained during the national surveillance of MW for HPAIV, were kindly provided by the National Wildlife Research Center, USDA/APHIS/WS (Fort Collins, CO, USA). The sequences of AIV isolates from poultry cases submitted to the USDA National Veterinary Services Laboratories (NVSL) in Ames, Iowa, from 2006 and 2011, were also included in the comparison. During that period, NVSL received suspect poultry cases from 17 states representing 13 species (Table 2). The majority of the cases were submitted from live bird markets. AIV isolates (*n* = 68) were obtained from three major poultry species: ducks, chickens, and turkeys (Table 2).

## 3. Results

### 3.1. Virus Isolation

Out of 1158 oropharyngeal-cloacal swabs tested, 87 AIV isolates (Table 3) were obtained, resulting in a 7.5% virus isolation success rate. These 87 LPAIV isolates represented oropharyngeal–cloacal samples from 31 sites in 27 counties in 17 states (California, Colorado, Delaware, Florida, Michigan, Mississippi, Montana, North Carolina, New Mexico, Ohio, Oregon, Pennsylvania, South Dakota, Utah, Washington, Wisconsin, and Wyoming). The isolates were obtained similarly from both live-captured-and-released and hunter-harvested non-mallard MW. Geographically, virus isolation-positive oropharyngeal-cloacal samples were collected from non-mallard MWs at sites within latitudes of 37.94° to 45.26°. Of these samples, only 10.5% (9/87) were from locations north of the 40° latitude.

A statistical comparison of VI success rates between the non-mallard and mallard MWs indicated that both MW types differed significantly (z = −3.88; *p* < 0.05). The total number of AIVs isolated from non-mallard MWs differed by species. The VI success rate among various non-mallard MWs is presented in Table 3. The majority of the AIV isolates (66/87, 75%) were obtained from four species: American black duck, American green-winged teal, American wigeon, and blue-winged teal (Table 3 and Table 4). Samples from blue-winged teal yielded the highest number of isolates (*n* = 38), followed by samples from American wigeon (*n* = 11), American black duck (*n* = 9), and American green-winged teal (*n* = 8). The virus isolations among non-mallard MWs also differed across flyways as follows: Atlantic 10.3% (9/87), Central 57.5% (50/87), Mississippi 17.2% (15/87), and Pacific 14.9% (13/87) (Table 4). However, pair-wise statistical comparisons indicated that only the following three flyway pairs differed significantly: Atlantic and Central (z = −7.57; *p* < 0.05); Central and Mississippi (z = 6.03; *p* < 0.05); and Central and Pacific (z = 6.51; *p* < 0.05). Physiologically, significantly more isolates were from male bird samples (56.3%; 49/87) than female bird samples, which represented 40.2% (35/87) of the isolates (z = 2.19; *p* < 0.05). The majority of the isolates were made from HY bird samples that represented 70.1% (61/87) of the total isolates, whereas AHY bird samples comprised 24.1% (21/87) of the isolates, indicating significantly higher isolations from HY birds (z = 7.16; *p* < 0.05). The sex of three and the age of five virus isolation-positive non-mallard MWs were not known.

### 3.2. Subtyping of LPAIV Isolates

The sequencing of 87 isolates revealed 21 HA and NA subtype combinations. The HA subtype on one of the isolates could not be confirmed (Table 5). The most commonly detected HA subtype was H4 (35/87, 40.2%), which was observed 1.5 and 2.5 times higher than the next common subtypes, H3 and H6, respectively. The most common NA subtype was N8 (40/87, 46.0%), which was observed 2.1 and 5.7 times higher than the next common subtypes, N6 and N5, respectively. The most commonly detected subtype combination was H3N8 (20/87, 23.0%), which was observed 1.25 times higher than both H4N6 and H4N8 subtypes (Table 5).

In comparison, 27 subtype combinations were identified among 149 isolates from mallard MWs [35]. The most common subtype combinations in mallards were H4N6 (34/149, 22.8%), H1N1 (23/149, 15.4%), H3N8 (17/149, 11.4%), H11N9 (8/149, 5.4%), H3N6 (7/149, 4.7%), H6N1 (7/149, 4.7%), H3N1 (6/149, 4.0%), and H10N7 (5/149, 3.4%).

Among the 68 poultry isolates (Table 3), the most common HA subtype was H6 (29/68, 42.6%), which was 1.4 and 4.8 times higher than the H4 and H3 subtypes, respectively. The most common NA subtype was N2 (24/68, 35.3%), which was observed 1.4 times higher than N6 and 2.4 times higher than N8. The most common subtype combinations were H4N6 (17/68, 25.0%) and H6N2 (17/68, 25.0%), which were observed 3.4 times higher than H6N1 (Table 6).

### 3.3. Geographic Distribution of LPAIV Subtypes

The flyway-wide distribution of various commonly detected AIV subtypes was as follows: Central Flyway: H3N8: 36.0% (18/50), H4N8: 30.0% (15/50), and H4N6: 18.0% (9/50); Mississippi Flyway: H6N2: 20.0% (3/15), H4N1: 13.0% (2/15), and H11N9: 13.0% (2/15); and Pacific Flyway: H4N6: 46.2% (6/13), and H6N5: 30.8% (4/13). In the Atlantic Flyway, nine AIV subtype combinations were found, but none of the subtypes was more common than the others (Table 4).

The subtypes H3N8, H4N6, and H6N5 were the most widely distributed geographically, all detected in three out of the four North American flyways. The subtype H3N8 was found in the Atlantic, Central, and Mississippi flyways, H4N6 in the Atlantic, Central, and Pacific flyways, and H6N5 in the Central, Mississippi, and Pacific flyways.

In comparison, mallard H4N6, H1N1, and H10N7 subtypes were the most widely distributed geographically and were found in all four North American flyways [35].

### 3.4. Temporal Patterns of LPAIVs

The virus isolation results demonstrated that the yearly prevalence of LPAIVs among non-mallard MWs varied with 3 isolates in 2006, 50 in 2007, 23 in 2008, 8 in 2009, 2 in 2010, and 1 in 2011.

The subtypes of AIV isolates from non-mallard MWs varied over the years, too. The yearly distribution for the H4N6 subtype spanned over three years (2007, 2008, 2010). Also, H3N8 and H6N5 AIVs were consecutively identified in 2007–2009, while H4N8 AIV was detected in 2007–2008. H10N3 AIV was isolated only from a sample collected in 2011. In addition to yearly variation, monthly variation in the detection of various AIV subtypes was also noted. For example, H4N8, H3N8, and H4N6 AIVs were commonly isolated from non-mallard MW samples collected during August–September, whereas H4N6, H6N5, and H3N8 AIVs were isolated from samples collected during October–December [35].

### 3.5. Phylogenetic Analyses

M gene sequencing of all isolates did not reveal any AIV of Eurasian lineage in the non-mallard MWs examined.

**Figure 1:**
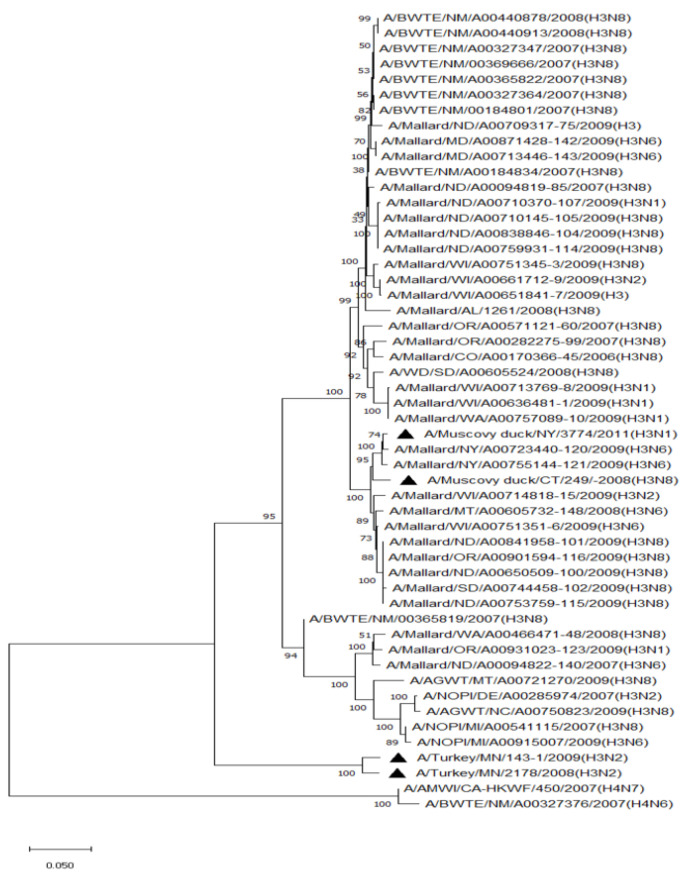
Phylogenetic relationship among H3 low pathogenic avian influenza virus isolates from non-mallard and mallard migratory waterbirds and poultry in the United States (2006–2011) based on HA sequences. Phylogenies were inferred using the maximum likelihood method with 100 bootstrap replicates for the viruses. Numbers at the nodes represent bootstrap values. Poultry isolates are indicated by a black triangle. Two H4 sequences were used to root the phylogenetic tree.

**Figure 2:**
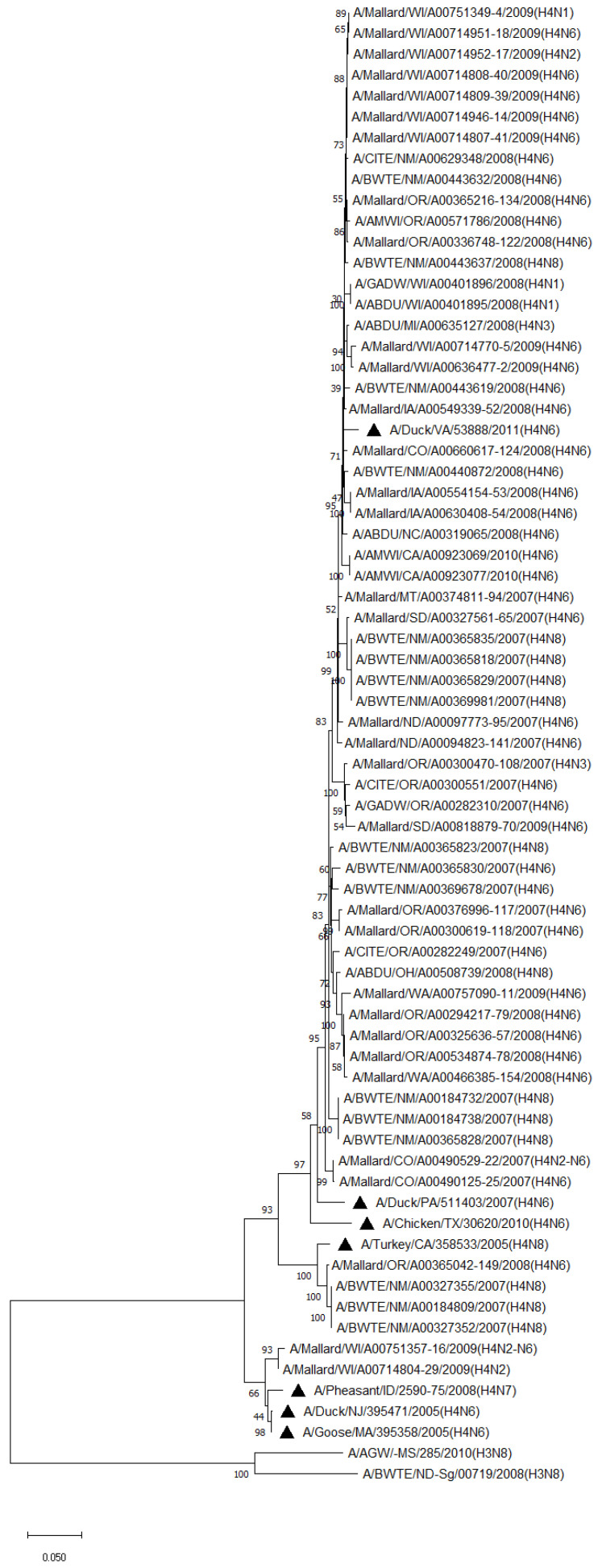
Phylogenetic relationships among H4 low pathogenic avian influenza virus isolates from non-mallard and mallard migratory waterbirds and poultry in the United States (2006–2011) based on HA sequences. Phylogenies were inferred using the maximum likelihood method with 100 bootstrap replicates for the viruses. Numbers at the nodes represent bootstrap values. Poultry isolates are indicated by a black triangle. Two H3 sequences were used to root the phylogenetic tree.

Phylogenetic analysis of HA genes of the most prevalent subtypes (H3 and H4) revealed a clear distinction between H3 and H4, as expected. H3 isolates shared 86.2–100% identity among non-mallard MWs, but no clustering between species or flyways within the same subtype was noted. The H3 AIVs of mallards shared 85.8–100% identity. The H3 isolates shared 85.5–99.5% identity between non-mallard and mallard MWs. H4 isolates shared 90.6–100% identity among non-mallard MWs, but no clustering between species or flyways within the same subtype was noted. The H4 AIVs of mallards shared 89.0–100% identity. The H4 isolates shared 89.3–99.7% identity between non-mallard and mallard MWs (Figure 1 and Figure 2).

**Figure 3:**
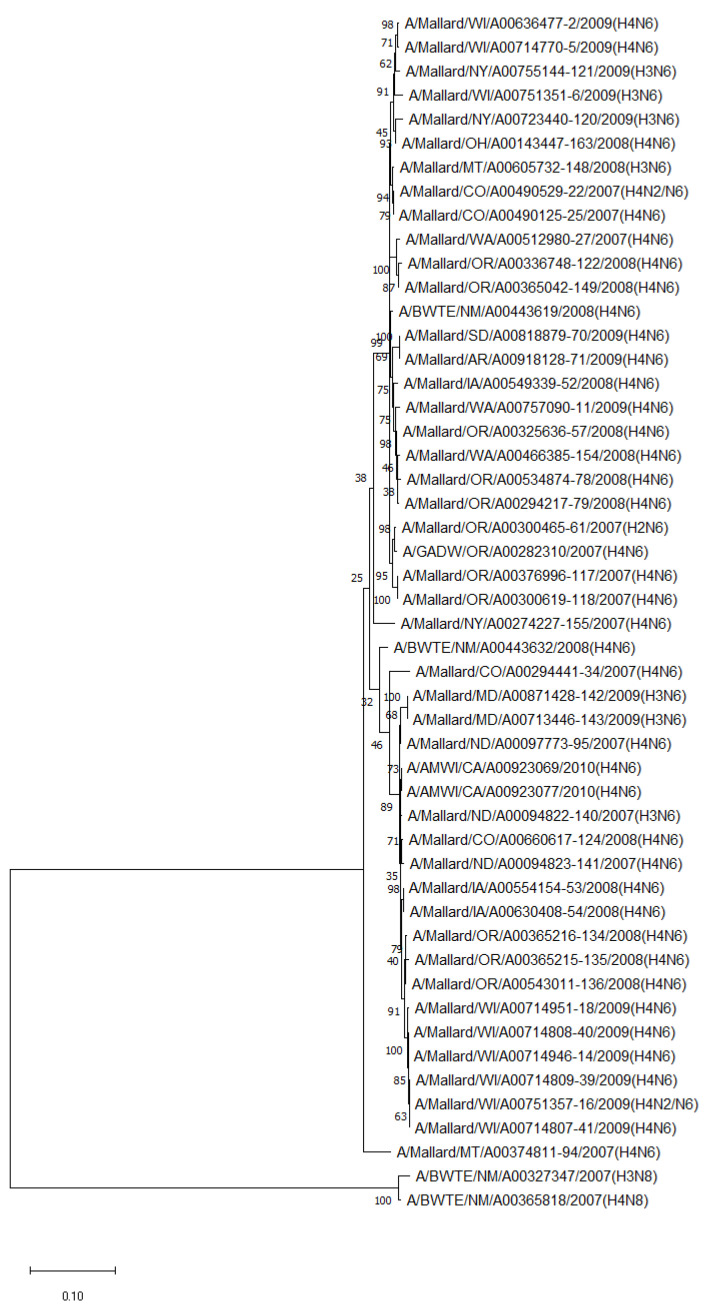
Phylogenetic relationship among N6 low pathogenic avian influenza virus isolates from non-mallard and mallard migratory waterbirds in the United States (2006–2011) based on NA sequences. Phylogenies were inferred using the maximum likelihood method with 100 bootstrap replicates for the viruses. Numbers at the nodes represent bootstrap values. Two N8 sequences were used to root the phylogenetic tree.

**Figure 4:**
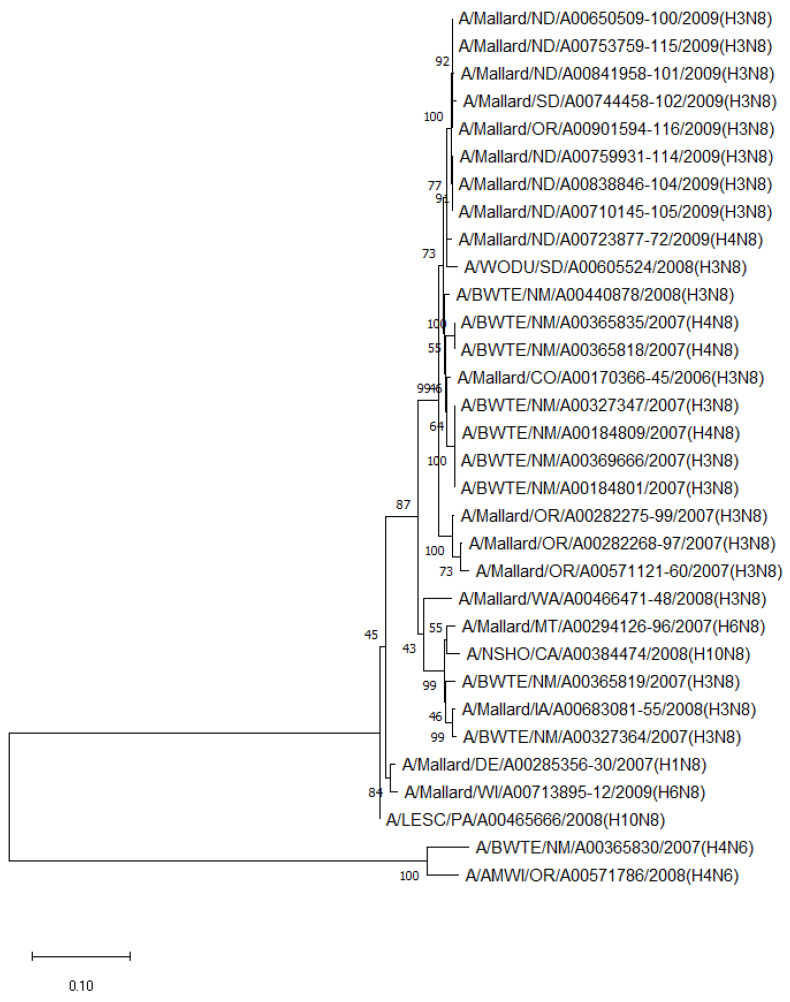
Phylogenetic relationship among N8 low pathogenic avian influenza virus isolates from non-mallard and mallard migratory waterbirds in the United States (2006–2011) based on NA sequences. Phylogenies were inferred using the maximum likelihood method with 100 bootstrap replicates for the viruses. Numbers at the nodes represent bootstrap values. Two N6 sequences were used to root the phylogenetic tree.

The phylogenetic analysis of NA genes of the most prevalent subtypes (N6 and N8) revealed a clear distinction between N6 and N8, as expected. Phylogenetic analyses of NA genes of the most prevalent subtypes (N6 and N8) also showed a clear distinction between subtypes (52.8–55.5% identity) but no clustering between species or flyways within the same subtype. Between non-mallard and mallard species, the N6 and N8 isolates shared 91.3–99.4% and 91.6–98.7% identity, respectively (Figure 3 and Figure 4).

When compared to the AIVs of poultry, common HA subtypes from non-mallard MWs showed 79.0–95.9% H3 and 89.5–98.4% H4 identity, respectively. The mallard H3 and H4 isolates shared 79.0–99.0% and 88.7–98.4% identity with poultry isolates, respectively. Overall, H3 and H4 isolates from both non-mallard and mallard MWs shared 79.0–99.0% and 88.7–98.4% identity with their counterparts in poultry, respectively. The comparison of NA sequences from both non-mallard MW and sequences from poultry revealed that N8 isolates shared 92.4–98.7% identity.

## 4. Discussion

The current study evaluated a subset of the AIV-positive OPC swab samples between 2006 and 2011, which were collected during the US nationwide HPAIV wild bird surveillance program [12]. Previous studies have indicated the key role of mallards in maintaining and spreading AIVs to poultry, yet the role of non-mallard MWs in maintaining AIVs remains largely unclear [36]. Therefore, this study focused on surveying and characterizing AIVs in non-mallard species of MWs.

The present study indicated geographic differences in the AIV positivity rate among non-mallard MWs across flyways. The percentage of positivity of AIVs from the Central flyway compared to that from the Atlantic, Mississippi, and Pacific flyways observed in this study is consistent with the report by Nallar et al. (2015) but differs from Gropper et al. (2014), who reported the highest positivity rate in the Pacific Flyway (2007–2008) and Mississippi Flyway (2009) [37,38]. While it could be argued that the flyway itself has little to no influence on AIV positivity rates, several ecological factors related to flyways, such as local environmental reservoirs and temperatures, are known to affect the AIV positivity rate and may have contributed to geographic differences in the positivity rate observed in this study [39]. Furthermore, the movement or aggregation patterns of MWs may also have contributed to the observed geographic differences, as reported previously [38].

Geographic differences were also noted in AIV subtypes among non-mallard MWs. For example, H3N8 AIVs were isolated more commonly from the birds in the Central Flyway. While H6N2 AIVs were more commonly isolated from the birds in the Mississippi Flyway, H4N6 AIVs were more commonly isolated from the birds in the Pacific Flyway. The geographic difference in the distribution of AIV subtypes noted in this study is consistent with the findings of Rejmanek et al. (2015) for MW [40]. Besides geographic differences, annual and seasonal fluctuations in AIV subtypes among non-mallard species were also observed in this study and agreed with previous studies reporting temporal differences among MWs [41,42].

Observed AIV subtypes varied among non-mallard species. The higher AIV subtype diversity in American black duck, American wigeon, gadwall, and northern pintail species observed in this study differs from Ferro et al. (2010), who found the greatest diversity of AIV subtypes in blue-winged teal, northern shoveler, and green-winged teal species [41]. One reason for the difference could be that even though conducted in a similar timeframe, Ferro and others (2010) studied AIVs in waterbirds only in the wintering grounds of Texas in 2009–2010, whereas the American black duck and American wigeon sampled in the present study represented nine other states (California, Colorado, Michigan, Montana, North Carolina, Ohio, Oregon, Washington, and Wisconsin). The AIV subtypes could be different in many of these northern states compared to Texas, which is a southern state. The present study also surveyed non-mallard MWs for AIV in four additional years (2006–2008, 2011) compared to the study by Ferro et al. (2010) [41].

The present study demonstrated differences in the virus isolation success rate among non-mallard MW species. The higher virus isolation success rate was noted in cinnamon teal, blue-winged teal, American wigeon, and American black duck species than other non-mallard MWs. Munster et al. (2007) also noted the highest prevalence of AIV in common teal [42]. Differences in virus isolation recovery rates observed among non-mallard MWs could be attributed to potential intrinsic physiological dissimilarities, such as foraging behaviors [15]. For example, cinnamon teal commonly dabbles, but also tips, when feeding on the bottom of a shallow pond. Cinnamon teal also likes to feed together (social feeding) [43]. Blue-winged teal species’ food is diverse as these are omnivorous and forage in shallow water that is no more than 8 inches deep [44]. Blue-winged teal normally dabbles but sometimes can dive to feed in shallow water [45]. American wigeon’s foraging pattern is known to differ with the habitat. For example, they graze while feeding on land. In water, they usually dabble but also upend and pick items from the surface of the water [46]. American black ducks forage by upending and dabbling [47]. Overall, the highest virus isolation success rate noted in dabbling ducks in this study is not unusual as these ducks are known to have a high prevalence of AIVs, perhaps due to their feeding behavior, which favors the ingestion of virus particles [48].

Blue-winged teal samples yielded a high number of AIV isolates (38 out of 128) in this study (Table 3). Such high virus isolation rates from blue-winged teal are consistent with the report by Ferro et al. (2010) and hold significance because these ducks play a part in the intercontinental spread of AIVs [41,49]. The oropharyngeal–cloacal samples from American wigeon and American black duck also resulted in a high number of isolates, suggesting their potential prominent role in AIV ecology, which is consistent with the reports by Hollander et al. (2019) and Ely et al. (2013) [50,51]. Furthermore, the American wigeon was one of the bird species that tested positive for HPAIV H5Nx during the AIV surveillance conducted from December 2014 to February 2017 in the US, suggesting the need to continually evaluate this species for any influenza activity [49]. However, our finding differs from Wilcox et al. (2011), who found comparatively low AIV prevalence in the Minnesota wigeon [6]. One reason for the difference between these two studies could be that Wilcox and others studied wigeons only in Minnesota, but our study represented six different states (California, Colorado, Montana, North Carolina, Oregon, and Washington), excluding the state of Minnesota. Together, these data confirm that cinnamon teal, blue-winged teal, American wigeon, and American black duck species should be a continued focus of AIV surveillance, as these dabbling ducks have a high prevalence of AIV consistent with USDA-USGS research [7].

The higher AIV isolations (*p* < 0.05) from male birds (56.3%) observed in this study are consistent with a report by Ip et al. (2008) and could be attributable to hormonal and behavioral differences between the male and female non-mallard MWs [52]. Male birds are likely to fight to defend their families, and in doing so, they can become exposed to AIVs orally during biting. Moreover, the dispersal behavior of males may place them at a higher risk of becoming infected with an AIV [51]. Besides sex, age, and immune status play a role in AIV positivity. A higher AIV isolation rate from hatch-year bird samples noted in the present study is consistent with previous studies and may be attributable to the virus-naïve status of hatch-year birds compared to after-hatch-year birds [53,54].

A difference in the virus isolation success rate was observed between non-mallard (~7.5%) and mallard (~12%) MWs. Non-mallard bird samples resulted in a significantly lower virus isolation success rate (*p* < 0.05) than mallard samples, even though approximately the same number of M-gene-positive samples were tested for both species [35]. The difference in the virus isolation success rate between non-mallards and mallards can be attributed to differences in species’ susceptibility to the virus, feeding behavior, physiologic demands, or immunological status. Non-mallard birds may be less permissive to AIV than mallards, leading to a low virus titer in these birds. Social actions, such as gregariousness, vagility, and dispersal characteristics, can also contribute to this difference. Mallards are known to have a high degree of sociability [55]. Such social behavior may influence viral exposure among birds, leading to higher virus recovery [48]. More importantly, the overall virus isolation success rate of ~10% from both mallard and non-mallard MWs suggests the need for improving virus survival during storage and transport.

It should be noted that the characterization of LPAIVs among MWs in this study was undertaken by the sequencing of isolates as per the US Interagency Strategic Plan for Avian Influenza Surveillance in Migratory Birds mandate. Since virus isolation can preferentially support some subtypes better than others [56], an unintentional bias may have been introduced in assessing LPAIV subtype diversity in MWs. Moreover, specific bird species may be more permissive to certain AIV subtypes [57]. To overcome such bias, PCR-based subtyping or next-generation sequencing directly on the oropharyngeal–cloacal swaps may yield a better picture of AIV subtypes circulating in various non-mallard MW species. Nevertheless, the present study highlighted common AIV subtypes circulating in various non-mallard MW species and indicated several MW species that could be focused on for AIV surveillance until new surveillance methods are developed and validated. Alternatively, better surveillance testing methods for subtyping, not relying on virus isolation, could provide an improved assessment of AIV prevalence and subtypes in MW. For example, Japanese scientists have used a PCR-based subtyping technique to support MW surveillance [58]. A similar approach could be adopted for the large-scale surveillance of MWs in the US [59]. Next-generation sequencing can also be used to obtain an unbiased or less-biased evaluation of AIV subtypes in a given sample [60].

Apart from the positivity rate, AIV subtype diversity in a given MW species informs us of its potential to sustain mixed infections, leading to new subtypes resulting from a reassortment among AIVs co-infecting a single host. Some subtypes, such as H3N8 and H4N6, were common in both mallard and non-mallard MWs surveyed in this study. The presence of genetically similar LPAIV subtypes in both species may be attributed to interspecies interactions between these species. Nonetheless, it should be noted that H1N1 was detected only in mallards during the USDA surveillance program [35], while H4N8 was common only in non-mallard MWs in the present study. However, other investigators have reported H1N1 in non-mallard MWs [61]. Several factors could account for subtype differences between species, such as differences in host susceptibility, virus tropism, replication, and immune evasion [62]. Such differences in AIV subtype diversity between non-mallard and mallard MWs suggest a need for studying AIV subtypes in both species categories.

The recovery of H11N9 from non-mallard MWs in this study is noteworthy because H11N9 LPAIV has seldom been reported from MW except for a recent recovery from a Mandarin duck in South Korea [63]. Since H11N9 is known to be common in shorebirds [64], interspecies interactions between non-mallard MWs and shorebirds might have contributed to the presence of H11N9 in non-mallard MWs [65].

Sequencing data of AIV isolates obtained during the current study based on M, HA, and NA genes of AIV did not suggest the introduction of an AIV of Eurasian lineage to North America through MWs between 2006 and 2011. There is still a need to monitor MWs for AIV as the intermingling of MWs from Eastern Siberia and Alaska in the Pacific and Central flyways provides opportunities for cross-continental transmission of AIVs [12]. Additionally, although a limited number of AIV sequences of US poultry isolates were used for comparison, the close sequence relatedness of MW isolates and poultry isolates for selected HA and NA subtypes [H3 (79.0–99.0%), H4 (88.7–98.4%), and N8 (92.4–98.7%)], suggests the potential role of MWs in introducing AIVs to poultry, corroborating other reports [4,5]. Therefore, the routine surveillance of MWs for AIV is warranted.

## 5. Conclusions

Overall, the higher AIV positivity rate noted in mallards supports the fact that AIV surveillance should focus on mallards if the surveillance program continues. Nevertheless, high AIV positivity rates noted in cinnamon teal, blue-winged teal, American wigeon, and American black duck species suggest that these non-mallard species should be a prime target of AIV surveillance efforts along with the mallards. American black duck and American wigeon, beside gadwall and northern pintail, harbored a variety of AIV subtypes, further signifying the importance of monitoring these birds concerning AIV diversity and reassortment. Additionally, our study data also suggest that male and hatch-year birds should continue to be the focus of AIV surveillance as their samples yield higher AIV positivity rates. While the present study provides insight into the LPAIV profile among non-mallard birds, these data should be compared with the more recent data to draw meaningful conclusions about species-wide AIV positivity patterns and subtype diversity over the years. Such comparison can shed light on AIV evolution in various MW species. Furthermore, monitoring AIVs in the reservoir species can inform research aimed at mitigating the impact of avian influenza on domestic poultry, as most poultry outbreaks in the US have been a result of the spillover from reservoir species.

## Figures and Tables

**Table 1:** Primer sequences for hemagglutinin genes designed/adapted in this study.

Subtype	Primer ID	Primer Sequence (5′-3′)	Purpose
H1	H1-943F	GGA GAA TGT CCC AAA TAT GTC	Sequencing
	H1-1250R	CCC ACT GCA GTG AAT TGA GTG	Sequencing
H2	H2-562R	ATT TAT CTG ATT TGA CAT ACT T	Sequencing
	H2-560R	GGA TGA TGT ACT CCC CAG ATG A	Sequencing
H3	H3-1233R	GGC ATT YGT TTT CTC GAT TAC YCG	Amplification
	H3-267R	GCA GAG CAT CCA CTA ATG TGC A	Sequencing
	H3-362R	TAA GGG TAA CAG TTG CTG	Sequencing
	H3-600Ra	CTC TTG GTT TGT GCT TGG ATG	Sequencing
	H3-869Fa	GAT GCA CCY ATT GAC ACA TG	Sequencing
	H3-869Fb	GAT GCA CCT ATT GAC ACA TG	Sequencing
	H3-869Fa	GAT GCA CCY ATT GAC ACA TG	Sequencing
	H3-869Fb	GAT GCA CCT ATT GAC ACA TG	Sequencing
	H3-810F	GGT AAC CTG ATC GCT CCT CG	Sequencing
	H3-1100R	GCT TGT CCT GTA CCT TCC GAG T	Sequencing
	H3-1150F	GGT ACA GGA CAA GCA GCA GAC	Sequencing
	H3-1233R	GGC ATT YGT TTT CTC GAT TAC YCG	Sequencing
	H3-1507F	GAC ATA TAC AGG GAC GAA GCA C	Sequencing
	H4-342Ra	GCA CAT CAA ATG GGT AAC AAG T	Sequencing
	H4-342Rb	GCA CAT CAA ATG GAT AAC AAG T	Sequencing
H4	H4-710 R	GCT GAT CCT ACC GCT TTG G	Sequencing
	H4-860F	GCG GTT CCT ATA GGA TCC TGC G	Sequencing
	H4-1343R	CTA ATG CCA CCA GCA ATT CAG C	Amplification/ Sequencing
	H4-1090Fa	GGT CTA ATC GAT GGT TGG TA	Sequencing
	H4-1090Fb	GGC AAG GAT TAA TTG ATG GGT	Sequencing
H6	H6-669Ra	AAT TCA TGC TTT CAG TCC CCA T	Sequencing
	H6-940F	CCT CTG TGG AWA GGA GAA TGC	Sequencing
	H6-1080R	GCC ATA CCA CCC ATC TAT CAT	Sequencing
	H6-1235R	TCG ACA GCT TCR AAT WGT GTG	Amplification/ Sequencing
H10	H10-971F	AAG AGC CTG TTG CTT GCT AC	Sequencing
	H10-250F	CTC CTG CTT GTG ACC TAC ACC	Sequencing
	H-10-260 F	GGA CTC CTG CTT GTG ACC TAC A	Sequencing
	H10-1153R	GAT CTA TAG CTG CCT GAG TAC	Amplification/ Sequencing
	H10-292R	GTA ACA GTA GGC AAT AGA ATT G	Sequencing
	H10-770R	GTG CTA TTA ATC CGC CAT TAT G	Sequencing
H11	H11-1185R	GGT CTA TTG CTT TCT GGG TTG	Amplification/ Sequencing
	H11-890F	CTC AAC TAA ATG CCA RTC CGA	Sequencing

**Table 2:** AIV subtypes of non-H5 and non-H7 detected from various poultry species across the United States (Fiscal Year 2006–2011) *.

State	Species **	AIV Subtype	No. Subtyped
Arkansas	Goose	H10N7	1
California	Turkey	H2N8	2
	Pekin ducks	H6N1,2,4 ^†^	3
	Chicken	H6N2	2
	Duck	H3N8	1
	Quail	H4N6; H6N2	2
	Unknown avian	H4N6	1
Delaware	Duck	H6N1	1
Florida	Chicken	H6N2	8
	Swan	H6N2	1
	Duck	H2N3; H6N2	2
Idaho	Duck	H2N9; H4N8	2
Indiana	Turkey	H3N2	1
Iowa	Chicken	H6N1,4 ^†^	2
Massachusetts	Duck	H11N2	1
Minnesota	Turkey	H4N2	1
	Turkey	H6N5	1
North Carolina	Chicken	H2N? ^‡^	1
New York	Duck	H6N8	1
Ohio	Duck	H4N2; H6N2	2
Oregon	Game birds	H3N8	2
Pennsylvania	Chicken	H4N6	1
	Turkey	H1N1	2
	Quail	H4N6	2
	Duck	H4N6	2
	Duck	H6N1,4 ^†^; H11N2	3
	Guinea fowl	H6N8	1
	Pheasant	H9N2	1
	Duck, Ostrich	H3N8; H4N8	2
Texas	Chicken	H4N6	7
	Chicken	H6N2	2
	Show ducks	H4N6	3
Washington	Duck	H6N1,4 ^†^; H10N7	3
Wisconsin	Turkey	H3N2	1
Total			68

* Adopted from the annual reports of the Committee on Transmissible Diseases of Poultry and Other Avian Species, 2006–2011. ** Common names of birds instead of specific species names were given in the report. ^†^ Mixed infection with more than one NA subtype according to the NVSL record. ^‡^ The question mark (?) after the N means that the neuraminidase subtype could not be identified.

**Table 3:** Virus isolation (VI) success rate in various non-mallard migratory waterbirds.

Species	No. of Samples Used in VI	No. of Isolates Obtained	Percent Success
American black duck (*Anas rubripes*)	97	9	9.3%
American green-winged teal (*Anas crecca carolinensis*)	103	8	7.8%
American wigeon (*Mareca americana*)	97	11	11.3%
Blue-winged teal (*Spatula discors*)	124	38	30.7%
Cinnamon teal (*Spatula cyanoptera*)	12	5	41.7%
Gadwall (*Mareca strepera*)	99	5	5.1%
Greater snow goose (*Anser caerulescens atlanticus*)	92	0	0.0%
Lesser scaup (*Aythya affinis*)	57	3	5.3%
Lesser snow goose (*Anser caerulescens*)	72	0	0.0%
Mute swan (*Cygnus olor*)	2	0	0.0%
Northern pintail (*Anas acuta*)	102	5	4.9%
Northern shoveler (*Spatula clypeata*)	100	2	2.0%
Ring-billed gull (*Larus delawarensis*)	25	0	0.0%
Ruddy turnstone (*Arenaria interpres*)	65	0	0.0%
Trumpeter swan (*Cygnus buccinator*)	2	0	0.0%
Tundra swan (*Cygnus columbianus*)	11	0	0.0%
Wood duck (*Aix sponsa*)	98	1	1.0%
Total	1158	87	7.5%

**Table 4:** Species-wide distribution of AIV subtypes in non-mallard migratory waterbirds across North American flyway.

Species	Flyway	Subtyping	No. Subtyped
American black duck	Atlantic	H4N6	1
	Mississippi	H2N9	1
	Mississippi	H4N1	1
	Mississippi	H4N3	1
	Mississippi	H4N8	1
	Mississippi	H6N5	1
	Mississippi	H10N8	1
	Mississippi	H11N9	2
American green-winged teal	Pacific	H10N7	1
	Central	H3N8	3
	Atlantic	H3N8	1
	Mississippi	H10N3	1
	Mississippi	H6N2	2
American wigeon	Central	H6N8	1
	Central	H10N3	1
	Central	H3N6	1
	Pacific	H6N5	4
	Pacific	H4N6	3
	Atlantic	H6N1	1
Blue-winged teal	Central	H3N8	14
	Central	H4N6	7
	Central	H4N8	15
	Central	H?N6 *	1
	Central	H10N7	1
Cinnamon teal	Pacific	H2N3	1
	Pacific	H4N6	2
	Central	H4N6	2
Gadwall	Mississippi	H4N1	1
	Pacific	H4N6	1
	Central	H6N1	1
	Mississippi	H6N2	1
	Central	H6N5	1
Lesser scaup	Atlantic	H6N3	1
	Atlantic	H10N8	1
	Atlantic	H11N9	1
Northern pintail	Atlantic	H3N2	1
	Atlantic	H12N4	1
	Mississippi	H3N6	1
	Mississippi	H3N8	1
	Central	H6N5	1
Northern shoveler	Atlantic	H8N4	1
	Pacific	H10N8	1
Wood duck	Central	H3N8	1
Total			87

* The question mark (?) after the H means that the hemagglutinin subtype could not be identified.

**Table 5:** Proportion (%) of non-H5 and H7 influenza A virus HA and NA subtypes among virus isolates detected in non-mallard migratory waterbirds for the years 2006–2011.

Subtype	N1	N2	N3	N4	N5	N6	N7	N8	N9	Total
H2			1.1 (1 *)						1.1 (1)	2.3 (2)
H3		1.1 (1)				2.3 (2)		23.0 (20)		26.4 (23)
H4	2.3 (2)		1.1 (1)			18.4 (16)		18.4 (16)		40.2 (35)
H6	2.3 (2)	3.4 (3)	1.1 (1)		8.0 (7)			1.1 (1)		16.1 (14)
H8				1.1 (1)						1.1 (1)
H10			2.3 (2)				2.3 (2)	3.4 (3)		8.0 (7)
H11									3.4 (3)	3.4 (3)
H12				1.1 (1)						1.1 (1)
H? ^†^						1.1 (1)				1.1 (1)
Total	4.6 (4)	4.6 (4)	5.7 (5)	2.3 (2)	8.0 (7)	21.8 (19)	2.3 (2)	46.0 (40)	4.6 (4)	

* Number of isolates classified to the given subtype. ^†^ The question mark (?) after the H means that the hemagglutinin subtype could not be identified.

**Table 6:** Proportion (%) of non-H5 and H7 influenza A virus HA and NA subtypes among virus isolates detected in poultry for the years 2006–2011 *.

Subtype	N1	N2	N3	N4	N5	N6	N7	N8	N9	N? ^‡^	Total
H1	2.9 (2 ^†^)										2.9 (2)
H2			1.5 (1)					2.9 (2)	1.5 (1)	1.5 (1)	7.4 (5)
H3		2.9 (2)						5.9 (4)			8.8 (6)
H4		2.9 (2)				25.0 (17)		2.9 (2)			30.9 (21)
H6	7.4 (5)	25.0 (17)		5.8 (4)	1.5 (1)			2.9 (2)			42.6 (29)
H9		1.4 (1)									1.5 (1)
H10							2.9 (2)				2.9 (2)
H11		2.9 (2)									2.9 (2)
Total	10.3 (7)	35.3 (24)	1.5 (1)	5.9 (4)	1.5 (1)	25.0 (17)	2.9 (2)	14.7 (10)	1.5 (1)	1.5 (1)	

* Adapted from the annual reports of the Committee on Transmissible Diseases of Poultry and Other Avian Species, 2006–2011. One mallard and seventeen H1N1pdm09 virus-like isolates were excluded from the data analysis for irrelevance. ^†^ Number of isolates classified to the given subtype. ^‡^ The question mark (?) after the N means that the neuraminidase subtype could not be identified.

## Data Availability

The sequence data presented in the study are available in the GenBank database. Data not presented in the article may be obtained by contacting the corresponding author.

## References

[1] Webster R.G., Bean W.J., Gorman O.T., Chambers T.M., Kawaoka Y. (1992). Evolution and ecology of influenza A viruses. Microbiol. Rev..

[2] Olsen B., Munster V.J., Wallensten A., Waldenström J., Osterhaus A.D., Fouchier R.A. (2006). Global patterns of influenza A virus in wild birds. Science.

[3] Spickler A.R., Trampel D.W., Roth J.A. (2008). The onset of virus shedding and clinical signs in chickens infected with high-pathogenicity and low-pathogenicity avian influenza viruses. Avian Pathol..

[4] Global Consortium for H5N8 and Related Influenza Viruses (2016). Role for migratory wild birds in the global spread of avian influenza H5N8. Science.

[5] Lee D.-H., Torchetti M.K., Hicks J., Killian M.L., Bahl J., Pantin-Jackwood M., Swayne D.E. (2018). Transmission Dynamics of Highly Pathogenic Avian Influenza Virus A(H5Nx) Clade 2.3.4.4, North America, 2014–2015. Emerg. Infect. Dis..

[6] Wilcox B.R., Knutsen G.A., Berdeen J., Goekjian V., Poulson R., Goyal S., Sreevatsan S., Cardona C., Berghaus R.D., Swayne D.E. (2011). Influenza-A Viruses in Ducks in Northwestern Minnesota: Fine Scale Spatial and Temporal Variation in Prevalence and Subtype Diversity. PLoS ONE.

[7] USDA-USGS (2015). Early Detection and Monitoring for Avian Influenzas of Significance in Wild Birds, a U.S. Interagency Strategic plan. https://www.aphis.usda.gov/animal_health/downloads/animal_diseases/ai/wild-bird-strategic-plan.pdf.

[8] Smallman-Raynor M., Cliff A.D. (2008). The geographical spread of avian influenza A (H5N1): Panzootic transmission (December 2003-May 2006); pandemic potential, and implications. Ann. Assoc. Am. Geogr..

[9] USDA APHIS (2022). Detections of Highly Pathogenic Avian Influenza. https://www.aphis.usda.gov/aphis/ourfocus/animalhealth/animal-disease-information/avian/avian-influenza/2022-hpai.

[10] USDA APHIS (2022). 2022 Confirmations of Highly Pathogenic Avian Influenza in Commercial and Backyard Flocks. https://www.aphis.usda.gov/livestock-poultry-disease/avian/avian-influenza/hpai-detections/commercial-backyard-flocks.

[11] Bevins S.N., Pedersen K., Lutman M.W., Baroch J.A., Schmit B.S., Kohler D., Gidlewski T., Nolte D.L., Swafford S.R., Deliberto T.J. (2014). Large-Scale Avian Influenza Surveillance in Wild Birds throughout the United States. PLoS ONE.

[12] USDA APHIS (2006). An Early Detection System for Highly Pathogenic H5N1 Avian Influenza in Wild Migratory Birds—U.S. Interagency Strategic Plan. https://www.ctahr.hawaii.edu/adap/avian_flu/AI_Manuals/US_national_wildbird_plan.pdf.

[13] Brand C.J. Surveillance Plan for the Early Detection of H5N1 Highly Pathogenic Avian Influenza Virus in Migratory Birds in the United States: Surveillance Year 2009. https://pubs.usgs.gov/gip/92/pdf/gip-92.pdf.

[14] Vittecoq M., Grandhomme V., Champagnon J., Guillemain M., Crescenzo-Chaigne B., Renaud F., Thomas F., Gauthier-Clerc M., van der Werf S. (2012). High Influenza A Virus Infection Rates in Mallards Bred for Hunting in the Camargue, South of France. PLoS ONE.

[15] Papp Z., Clark R.G., Parmley E.J., Leighton F.A., Waldner C., Soos C. (2017). The ecology of avian influenza viruses in wild dabbling ducks (Anas spp.) in Canada. PLoS ONE.

[16] Azeem S. (2020). Avian Influenza Virus in Migratory Wild Birds and Poultry in the United States. Ph.D. Dissertation.

[17] Deliberto T.J., Swafford S.R., Nolte D.L., Pedersen K., Lutman M.W., Schmit B.B., Baroch J.A., Kohler D.J., Franklin A. (2009). Surveillance for highly pathogenic avian influenza in wild birds in the USA. Integr. Zool..

[18] Boere G.C., Stroud D.A., Boere G.C., Galbraith C.A., Stroud D.A. (2006). The flyway concept: What it is and what it isn’t. Waterbirds around the World.

[19] Swayne D.E., Senne D.A., Beard C.W., Swayne D.E., Gillson J.R., Jackwood M.W., James E.P., Willie M.R. (1998). Avian Influenza. A Laboratory Manual for the Isolation and Identification of Avian Pathogens.

[20] Krauss S., Walker D., Webster R.G., Kawaoka Y., Neumann G. (2012). Influenza virus isolation. Influenza Virus: Methods and Protocols, Methods in Molecular Biology.

[21] Senne D.A., Louise D.-Z., Swayne D.E., Glisson J.R., Peason J.E., Reed W.M., Jackwood M.W. (2008). Virus propagation in embryonating eggs. A Laboratory Manual for the Isolation: Identification and Characterization of Avian Pathogens.

[22] Killian M.L., Spackman E. (2008). Hemagglutination assay for the avian influenza virus. Avian Influenza Virus.

[23] Spackman E., Spackman E. (2014). Avian Influenza virus detection and quantitation by real-time RT-PCR. Animal Influenza Virus.

[24] Sanger F., Nicklen S., Coulson A.R. (1977). DNA sequencing with chain-terminating inhibitors. Proc. Natl. Acad. Sci. USA.

[25] Schweiger B., Zadow I., Heckler R., Timm H., Pauli G. (2000). Application of a fluorogenic PCR assay for typing and subtyping of influenza viruses in respiratory samples. J. Clin. Microbiol..

[26] Hoffmann E., Stech J., Guan Y., Webster R.G., Perez D.R. (2001). Universal primer set for the full-length amplification of all influenza A viruses. Arch. Virol..

[27] Chang K.H., Park J.H., Song M.-S., Oh T.-K., Kim S.-Y., Kim C.-J., Kim H.-G., Sung M.-H., Han H.-S., Hahn Y.-S. (2008). Development of multiplex RT-PCR assays for rapid detection and subtyping of influenza type A viruses from clinical specimens. J. Microbiol. Biotechnol..

[28] Lee M.S., Chang P.C., Shien J.H., Cheng M.C., Shieh H.K. (2001). Identification and subtyping of avian influenza viruses by reverse transcription-PCR. J. Virol. Methods..

[29] Duitama J., Kumar D.M., Hemphill E., Khan M., Mǎndoiu I.I., Nelson C.E. (2009). PrimerHunter: A primer design tool for PCR-based virus subtype identification. Nucleic Acids Res..

[30] Piaggio A.J., Shriner S.A., VanDalen K.K., Franklin A.B., Anderson T.D., Kolokotronis S.O. (2012). Molecular surveillance of low pathogenic avian influenza viruses in wild birds across the United States: Inferences from the hemagglutinin gene. PLoS ONE.

[31] Zohari S., Metreveli G., Kiss I., Belák S., Berg M. (2010). Full genome comparison and characterization of avian H10 viruses with different pathogenicity in Mink (*Mustela vison*) reveals genetic and functional differences in the non-structural gene. Virol. J..

[32] Lang A.S., Kelly A., Runstadler J.A. (2008). Prevalence and diversity of avian influenza viruses in environmental reservoirs. J. Gen. Virol..

[33] Chander Y., Jindal N., Stallknecht D.E., Sreevatsan S., Goyal S.M. (2010). Full length sequencing of all nine subtypes of the neuraminidase gene of influenza A viruses using subtype specific primer sets. J. Virol. Methods.

[34] Kumar S., Stecher G., Li M., Knyaz C., Tamura K. (2018). MEGA X: Molecular evolutionary genetics analysis across computing platforms. Mol. Biol. Evol..

[35] Baroch J. National Wildlife Research Center: Fort Collins, CO, USA, 2024, unpublished.

[36] Nishiura H., Hoye B., Klaassen M., Bauer S., Heesterbeek H. (2009). How to find natural reservoir hosts from endemic prevalence in a multi-host population: A case study of influenza in waterfowl. Epidemics.

[37] Nallar R., Papp Z., Epp T., Leighton F.A., Swafford S.R., DeLiberto T.J., Dusek R.J., Ip H.S., Hall J., Berhane Y. (2015). Demographic and Spatiotemporal Patterns of Avian Influenza Infection at the Continental Scale, and in Relation to Annual Life Cycle of a Migratory Host. PLoS ONE.

[38] Groepper S.R., Deliberto T.R., Vrtisja M.P., Pedersen K., Swafford S.R., Hygnstrom S.E. (2014). Avian influenza virus prevalence in migratory waterfowl in the United States, 2007–2009. Avian Dis..

[39] Gorsich E.E., Webb C.T., Merton A.A., Hoeting J.A., Miller R.S., Farnsworth M.L., Swafford S.R., DeLiberto T.J., Pedersen K., Franklin A.B. (2021). Continental scale dynamics of avian influenza in U.S. waterfowl are driven by demography, migration, and temperature. Ecol. Appl..

[40] Rejmanek D., Hosseini P.R., Mazet J.A.K., Daszak P. (2015). Evolutionary dynamics and global diversity of influenza A virus. J. Virol..

[41] Ferro P.J., Budke C.M., Peterson M.J., Cox D., Roltsch E., Merendino T., Nelson M., Lupiani B. (2010). Multiyear Surveillance for Avian Influenza Virus in Waterfowl from Wintering Grounds, Texas Coast, USA. Emerg. Infect. Dis..

[42] Munster V.J., Baas C., Lexmond P., Waldenstrom J., Wallensten A., Fransson T., Rimmelzwaan G.F., Beyer W.E., Schutten M., Olsen B. (2007). Spatial, temporal, and species variation in prevalence of influenza A viruses in wild migratory birds. PLoS Pathog..

[43] Gammonley J., Poole A. (2020). Cinnamon Teal (*Spatula cyanoptera*), version 1.0. The Birds of North America.

[44] Gammonley J.H., Fredrickson L.H. (1995). 13.1.8. Life History and Management of the Blue-Winged Teal. Waterfowl Manag. Handbook 39. https://digitalcommons.unl.edu/icwdmwfm/39.

[45] Swanson G.A., Meyer M.I., Serie J.R. (1974). Feeding ecology of breeding blue-winged teals. J. Wildl. Manag..

[46] Mini A.E., Harrington E.R., Rucker E., Dugger B.D., Mowbray T.B., Poole A.F. (2020). American Wigeon (*Moreca americana*) version 1.0. Birds of North America.

[47] Bennett M.J. Comparison of the diet, feeding behavior, and habitat use of mallards (*Anas platyrhynchos*) and Black Ducks (*Anas rubripes*). Biology Honors Paper. 1987, 18. https://digitalcommons.conncoll.edu/biohp/18.

[48] Jourdain E., Gunnarsson G., Wahlgren J., Latorre-Margalef N., Bröjer C., Sahlin S., Svensson L., Waldenström J., Lundkvist A., Olsen B. (2010). Influenza Virus in a Natural Host, the Mallard: Experimental Infection Data. PLoS ONE.

[49] USDA (2017). Surveillance Plan for Highly Pathogenic Avian Influenza in Wild Migratory Birds in the United States. https://www.aphis.usda.gov/animal_health/downloads/animal_diseases/ai/2017-hpai-surveillance-plan.pdf.

[50] Hollander L.P., Fojtik A., Kienzle-Dean C., Davis-Fields N., Poulson R.L., Davis B., Mowry C., Stallknecht D.E. (2018). Prevalence of Influenza A Viruses in Ducks Sampled in Northwestern Minnesota and Evidence for Predominance of H3N8 and H4N6 Subtypes in Mallards, 2007–2016. Avian Dis..

[51] Ely C.R., Hall J.S., Schmutz J.A., Pearce J.M., Terenzi J., Sedinger J.S., Ip H.S. (2013). Evidence that Life History Characteristics of Wild Birds Influence Infection and Exposure to Influenza A Viruses. PLoS ONE.

[52] Ip H.S., Flint P.L., Franson J.C., Dusek R.J., Derksen D.V., E Gill R., Ely C.R., Pearce J.M., Lanctot R.B., Matsuoka S.M. (2008). Prevalence of Influenza A viruses in wild migratory birds in Alaska: Patterns of variation in detection at a crossroads of intercontinental flyways. Virol. J..

[53] Krauss S., Walker D., Pryor S.P., Niles L., Chenghong L., Hinshaw V.S., Webster R.G. (2004). Influenza A viruses of migrating wild aquatic birds in North America. Vector Borne Zoonotic Dis..

[54] Hinshaw V.S., Webster R.G., Turner B. (1980). The perpetuation of orthomyxoviruses and paramyxoviruses in Canadian waterfowl. Can. J. Microbiol..

[55] Bahl J., Krauss S., Kühnert D., Fourment M., Raven G., Pryor S.P., Niles L.J., Danner A., Walker D., Mendenhall I.H. (2013). Influenza A Virus Migration and Persistence in North American Wild Birds. PLoS Pathog..

[56] Lindsay L.L., Kelly T.R., Plancarte M., Schobel S., Lin X., Dugan V.G., Wentworth D.E., Boyce W.M. (2013). Avian Influenza: Mixed Infections and Missing Viruses. Viruses.

[57] Alexander D.J. (2000). A review of avian influenza in different bird species. Vet. Microbiol..

[58] Tsukamoto K., Javier P.C., Shishido M., Noguchi D., Pearce J., Kang H.M., Jeong O.M., Lee Y.J., Nakanishi K., Ashizawa T. (2012). SYBR green-based real-time reverse transcription-PCR for typing and subtyping of all hemagglutinin and neuraminidase genes of avian influenza viruses and comparison to standard serological subtyping tests. J. Clin. Microbiol..

[59] Azeem S., Guo B., Sun D., Killian M.L., Baroch J.A., Yoon K.J. (2022). Evaluation of PCR-based hemagglutinin subtyping as a tool to aid in surveillance of avian influenza viruses in migratory wild birds. J. Virol. Methods.

[60] Park H.C., Shin J., Cho S.M., Kang S., Chung Y.J., Jung S.H. (2020). PAIVS: Prediction of avian influenza virus subtype. Genomics Inform..

[61] Ramey A.M., Reeves A.B., TeSlaa J.L., Nashold S., Donnelly T., Bahl J., Hall J.S. (2016). Evidence for common ancestry among viruses isolated from wild birds in Beringia and highly pathogenic intercontinental reassortant H5N1 and H5N2 influenza A viruses. Infect. Genet. Evol..

[62] Verhagen J.H., Lexmond P., Vuong O., Schutten M., Guldemeester J., Osterhaus A.D.M.E., Elbers A.R.W., Slaterus R., Hornman M., Koch G. (2017). Discordant detection of avian influenza virus subtypes in time and space between poultry and wild birds; Towards improvement of surveillance programs. PLoS ONE.

[63] Tuong H.T., Nguyen N.M., Sung H.W., Yun K.J., Park H., Yeo S.-J. (2020). Genetic characterization of avian influenza A (H11N9) virus isolated from mandarin ducks in South Korea in 2018. Viruses.

[64] Hurtado R., Fabrizio T., Vanstreels R.E.T., Krauss S., Webby R.J., Webster R.G., Durigon E.L. (2015). Molecular Characterization of Subtype H11N9 Avian Influenza Virus Isolated from Shorebirds in Brazil. PLoS ONE.

[65] Postnikova Y., Treshchalina A., Boravleva E., Gambaryan A., Ishmukhametov A., Matrosovich M., Fouchier R.A.M., Sadykova G., Prilipov A., Lomakina N. (2021). Diversity and reassortment rate of influenza A viruses in wild ducks and gulls. Viruses.

